# Secretion of IL-1β From Monocytes in Gout Is Redox Independent

**DOI:** 10.3389/fimmu.2019.00070

**Published:** 2019-01-29

**Authors:** Ben M. Alberts, Connor Bruce, Kolitha Basnayake, Pietro Ghezzi, Kevin A. Davies, Lisa M. Mullen

**Affiliations:** ^1^Clinical and Experimental Medicine, Brighton and Sussex Medical School, Brighton, United Kingdom; ^2^Sussex Kidney Unit, Royal Sussex County Hospital, Brighton, United Kingdom

**Keywords:** IL-1β, gout, chronic kidney disease, NLRP3 inflammasome, antioxidant capacity, redox regulation, reactive oxygen species

## Abstract

The pro-inflammatory cytokine interleukin-1β (IL-1β) plays important roles in immunity but is also implicated in autoimmune disease. The most well-established mechanism of IL-1β secretion is via activation of the NOD-like receptor family pyrin domain containing-3 (NLRP3) inflammasome which requires an initial priming signal followed by an activating signal. However, the precise mechanism by which the inflammasome is activated remains unclear. The role of reactive oxygen species (ROS) in this process is contradictory, with some studies suggesting that ROS are crucial while others describe opposite effects. In this study, we evaluated the effects of oxidative stress on IL-1β secretion. Gout is a disease driven solely by IL-1β secretion in response to monosodium urate (MSU) crystals which form during periods of hyperuricemia and thus presents an opportunity to study factors contributing to IL-1β secretion. Sera and monocytes were isolated from patients with gout to determine whether differences in antioxidant status could explain the susceptibility of these individuals to gout attacks. In addition, sera and monocytes were collected from patients with chronic kidney disease (CKD) for comparison as this condition is associated with high levels of oxidative stress and disturbances in serum uric acid levels. There were differences in some aspects of antioxidant defenses in gout patients and these were mainly due to higher serum uric acid. Monocytes from gout patients were more responsive to priming, but not activation, of the NLRP3 inflammasome. However, expression of the components of the NLRP3 inflammasome were unaffected by priming or activation of the inflammasome, nor were these expression levels differentially regulated in gout patients. Inhibition of ROS by N-Acetyl Cysteine inhibited TLR2-induced priming of the NLRP3 inflammasome, but had no effect on MSU-induced activation. Together these findings demonstrate that oxidative stress only affects priming of the NLRP3 inflammasome but does not influence activation.

## Introduction

Pro-inflammatory cytokines play important roles in protecting against infection but are also implicated in autoimmune and metabolic disease (1, 2). Prototypical pro-inflammatory cytokines include tumor necrosis factor-α (TNFα), interleukin-6 (IL-6), and IL-1β. The resounding success of the anti-TNF and anti-IL-6 therapies for conditions such as Rheumatoid arthritis (RA) (3, 4) and SLE (5), respectively, has caused renewed interest in understanding how, why and when these potent molecules are secreted in health and disease. The mechanism of secretion of IL-1β has been a long-standing conundrum in the field of cytokine biology. Identification of the NOD-like receptor family pyrin domain containing-3 (NLRP3) inflammasome as an essential component of IL-1β processing as first described by Martinon et al. (6) was a critical step in elucidating the mechanism by which intracellular pro-IL-1β is processed for subsequent secretion where it contributes to inflammatory responses.

The NLR family pyrin domain containing 3 (NLRP3) inflammasome is a tripartite intracellular receptor consisting of three proteins, NLRP3, ASC, and pro-caspase-1 which oligomerize to form a cytosolic complex. Canonical NLRP3 inflammasome activation involves two distinct signals, an initial “priming” signal that increases transcription of pro-IL-1β and NLRP3 followed by a second signal to induce oligomerization. NLRP3 is activated by a wide array of stimuli, such as extracellular ATP (7), asbestos (8), and the influenza virus (9), and is involved in the pathogenesis of many diseases (10).

Despite decades of research on this topic, the precise molecular mechanisms by which the inflammasome is activated are still unclear. One hypothesis, which has been the subject of numerous studies, is that increased oxidative stress may be involved in this process. Oxidative stress is defined as an imbalance between the levels of reactive oxygen species (ROS) and the antioxidant defenses that regulate these levels. ROS are important signaling molecules (11), but if present in very high concentrations can damage biological molecules including proteins, lipids, and nucleic acids (12). Perhaps unsurprisingly then, oxidative stress has been implicated in a myriad of human diseases (13).

Many studies have presented evidence for the involvement of ROS, and by extension oxidative stress, in activation of the NLRP3 inflammasome (8, 14). Furthermore, NLRP3 is itself redox sensitive (15–18). However, the role of ROS and oxidative stress is far from clear with many conflicting lines of evidence and contradictory results (8, 19). This suggests it is unlikely that there is a simple mechanistic relationship between ROS and oligomerization of NLRP3; rather, any such relationship is more likely to be subtle and involve oxidative stress in its entirety rather than simply an increase in ROS.

Although IL-1β production occurs in many inflammatory conditions, gout is unique in that it is primarily driven by this cytokine. Gout is caused by precipitation of uric acid from the blood into insoluble crystals of monosodium urate (MSU) that can accumulate in joints and activate the NLRP3 inflammasome resulting in secretion of IL-1β. Hyperuricemia is the greatest single risk factor for developing gout and yet intriguingly, only about 10% of hyperuricemic individuals ever have symptoms of gout (20, 21). Furthermore the presence of MSU crystals within joints does not necessarily precipitate the development of an inflammatory attack (22, 23). Clearly there are other, as yet unknown, factors that determine whether production of IL-1β occurs as a result of hyperuricemia.

Thus, gout presents a unique opportunity to investigate the factors that contribute to IL-1β production, and in particular the role of oxidative stress on this process, as uric acid has both pro-and anti-oxidant effects depending on location and context (24, 25). This dual and paradoxical nature of uric acid means that long-term exposure of blood monocytes to high levels of uric acid could result in either increased oxidative stress or a compensatory increase in antioxidant defenses, either of which could impact on IL-1β production (26, 27). The aims of this study are to investigate the role of oxidative stress on IL-1β production in primary human monocytes isolated from people with a history of gout by focusing on intracellular and extracellular antioxidant defenses rather than on the more commonly studied ROS. Peripheral blood monocytes from these patients will be analyzed to determine whether changes occur in the molecular machinery involved in the production and/or processing of IL-1β in these cells as a result of exposure to high concentrations of uric acid and potentially altered extracellular redox state. These patients have by definition a history of hyperuricemia, but do not have acute inflammation at the time of blood sampling that could confound the analyses performed.

Production of IL-1β from monocytes isolated from patients with chronic kidney disease (CKD) or with RA will also be studied for comparison. Oxidative stress is implicated in the pathophysiology in both of these conditions (28, 29). CKD patients in particular have perturbations in serum uric acid which could alter the levels of oxidative stress. However, neither of these disease is primarily driven by IL-1β thus providing useful comparisons to gout.

We also measured the activity or gene expression of antioxidant enzymes previously associated with regulation of the inflammatory response (30, 31). SOD2 is the mitochondrial isozyme of superoxide dismutase (SOD) and detoxifies superoxide radicals produced by the respiratory chain in the mitochondria (32). Thioredoxin reductase 1 (TXNRD) reduces the oxidized form of thioredoxin to the reduced form that reduces a wide range of proteins and, in association with peroxiredoxins (thioredoxin peroxidase), detoxifies hydrogen peroxide (33). Glutathione peroxidase 3 (Gpx3) is an extracellular peroxidase that also detoxifying hydrogen peroxide, but uses reduced glutathione as the electron donor (34) and extracellular superoxide dismutase (ecSOD) is the extracellular isozyme of SOD, detoxifying superoxide radicals in extracellular spaces (35).

## Materials and Methods

### Reagents

Cell culture media and fetal bovine serum (FBS) were purchased from Sigma-Aldrich (Gillingham, UK). Penecillin/streptomycin were purchased from Thermo Fischer Scientific (East Grinstead, UK). MSU crystals were purchased from Invivogen (San Diego, CA). Qiazol, QuantiTect reverse transcription kit and QuantiFast-SYBR green PCR kit were from Qiagen (Manchester, UK).

### Isolation of Primary Human Monocytes

Monocytes were isolated from 30 ml of whole blood collected from healthy volunteers (*n* = 52), or patients with a history of gout (*n* = 50), CKD (*n* = 42), or RA (*n* = 36) after giving informed written consent (NRES reference: 15/NS/0083). Gout patients were defined as individuals who had suffered from an inflammatory arthritic attack clinically diagnosed as gout. Many of the gout patients were prescribed allopurinol or benzbromarone to be used daily, or colchicine or naproxen to use as required. CKD patients were undergoing triweekly haemodialysis and blood was taken prior to starting haemodialysis. RA patients were identified and recruited from rheumatology clinics. Peripheral blood mononuclear cells (PBMCs) were isolated from whole blood by density gradient separation using lympholyte-H cell separation media (VH Bio, UK). Monocytes were then isolated from the PBMCs using CD14+ magnetic beads (Miltenyi Biotec, Woking, UK) yielding a population of monocytes with >95% purity. For stimulation experiments, cells were seeded at 4 × 10^4^ cells/well in 384-well tissue culture plates and stimulated for 18 h in RPMI-1640 media supplemented with 5% (v/v) FBS and streptomycin/penicillin (100 μg/mL and 100 U/mL, respectively) containing Pam3 +/– MSU crystals. A separate 10 mL sample of blood was collected from each donor in spray-coated silica tubes (Becton-Dickinson, Plymouth, UK) and allowed to clot for at least 1 h. After clotting, samples were centrifuged at 1,300 × g for 10 min and serum collected and stored at −80°C until future use.

Primary human monocytes were also isolated from single donor plateletphoresis residues obtained from the North London Blood Transfusion Center (United Kingdom). PBMCs were isolated by density gradient separation using lympholyte-H cell separation media (VH Bio, UK) followed by isolation of monocytes by Percoll (Sigma-Aldrich) density gradient centrifugation (36, 37). For analysis of intracellular gene expression and total antioxidant capacity (TAC) monocytes were seeded at around 1.5–2.5 × 10^6^ cells per well in 24 well-plates and preincubated for 16 h with 30 mg/dl uric acid. Following preincubation, cells were stimulated for 6 h with or without Pam3 (100 ng/mL) in the presence of 30 mg/dL uric acid.

### Quantification of IL-1β

IL-1β in cell supernatants was measured by ELISA using matched anti-human IL-1β antibodies purchased from R&D systems (Oxon, UK).

### RNA Extraction, Reverse Transcription and Absolute RT-qPCR

Depending on monocyte yield, 0.5–1.6 × 10^6^ monocytes were lysed in 0.5 mL of Qiazol and RNA was extracted using an RNeasy kit (Qiagen) and reverse transcribed to cDNA using the QuantiTect reverse transcription kit (Qiagen) according to the manufacturer's instructions. Quantitative real-time RT-PCR (qPCR) assays for absolute quantification of gene expression were purchased from qStandard (London, UK). Copy numbers for NLRP3 (*NLRP3*), pro-caspase-1 (*CASP1*), pro-IL-1β (*IL1B*), ASC-1 (*PYCARD*), superoxide dismutase-2 (*SOD2*), and thioredoxin reductase-1 (*TXNRD1*) were determined by qPCR using QuantiFast SYBR green PCR kit on a Stratagene Mx3000 thermocycler (Agilent Technologies, UK) or a Rotorgene thermocycler (Qiagen) under the following thermocycling program; 40 cycles of 95°C for 15 s and 60°C for 30 s with an initial cycle of 95°C for 15 min. Beta-2 Microglobulin (*B2M*) and Ribosomal Protein L32 (*RPL32*) were selected as reference genes following initial experiments confirming their stability and were used for normalization of gene of interest (GOI) copy numbers. Primer sequences for all genes are shown in Table 1. Copy number for each GOI and reference gene were calculated from a standard curve of known copy numbers for each gene run on the same plate. Copy numbers were normalized to *B2M* and *RPL32* (38).

**Table 1:** Forward and reverse primer sequences for genes measured by qPCR.

**Gene**	**Forward**	**Reverse**
*B2M*	5′-ctctctctttctggcctggag-3′	5′-acccagacacatagcaattcag-3′
*RPL32*	5′-catctccttctcggcatcat-3′	5′-accctgttgtcaatgcctct-3′
*CASP1*	5′-atgcctgttcctgtgatgtgg-3′	5′-ctcttcacttcctgcccacaga-3′
*IL1B*	5′-gtaatgacaaaatacctgtggccttg-3′	5′-tttgggatctacactctccagc-3′
*NLRP3*	5′-gagatgagccgaagtggggttc-3′	5′-gcttctcacgtactttctgtacttct-3′
*PYCARD*	5′-gctaacgtgctgcgcgacat-3′	5′-ccactcaacgtttgtgaccct-3′
*SOD2*	5′-aagtaccaggaggcgttgg-3′	5′-cgtcagcttctccttaaacttgtc-3′
*TXNRD1*	5′-tatgggcaatttattggtcctcaca-3′	5′-gctccaacaaccagggtcttac-3′

### Serum Measurements

Serum uric acid concentrations were measured using an Amplex Red-based uric acid/uricase assay kit (ThermoFisher, Loughborough, UK) according to the manufacturer's instructions. Activity of serum glutathione peroxidase (GPx) and SOD were determined using HT glutathione peroxidase and HT superoxide dismutase assay kits (Trevigen, Maryland, US), respectively, according to the manufacturer's instructions. GPx activity was measured as rate of NADPH decrease per minute over a 10 min period. SOD activity was measured as percentage inhibition of the rate of WST-1 reduction per minute over a 10 min period. Total serum protein concentrations were determined using BCA protein assay (ThermoFisher) according to the manufacturer's instructions and used to calculate activity per mg protein. Serum TAC was determined using an Antioxidant assay kit (Abnova, UK) according to the manufacturer's instructions.

### Intracellular Total Antioxidant Capacity

For measurement of intracellular TAC capacity, monocytes were lysed in 50–100 μL of NP40 lysis buffer (10 mM Tris-HCl, 150 mM NaCl, 1 mM EGTA, 1% NP-40, pH 7.4). TAC was measured as above, and normalized to the protein content of the sample.

### Measurement of ROS

ROS generation was detected using the ROS-Glo™ assay (Promega, Southampton, UK) according to the manufacturer's instructions (39). Briefly, cells were stimulated for 2 h with Pam3, MSU, or soluble uric acid in the presence of 25 μM ROS-Glo H_2_O_2_ substrate. Stimulations were completed in serum-free Optimem cell culture media (Gibco, ThermoFisher). After 2 h incubation at 37°C and 5% CO_2_, ROS levels were determined following a 20 min incubation with the ROS-Glo™ detection reagent. Luminescence was measured by spectrophotometry (Biotek Synergy HT).

N-acetyl-L-cysteine (NAC) was used in some experiments to inhibit ROS in primary human monocytes. For these experiments, cells were preincubated for 1 h with NAC and then stimulated for 6 h with Pam3 ± MSU crystals. NAC concentration was maintained during the 6 h stimulation. ROS levels were determined by ROS-Glo™ and IL-1β secretion by ELISA.

### Assessment of Cell Viability

Cell viability was assessed at the end of each experiment using the Cell-Titer Glo™ assay (Promega) according to the manufacturer's instructions.

### Statistical Analysis

Differences between groups were assessed using non-parametric Kruskall–Wallis one-way analysis of variance (ANOVA) followed by Dunn's multiple comparison test or by Mann–Whitney tests. The level of significance was set at *P* < 0.05. All analyses were conducted using GraphPad Prism version 7 (Graphpad software, San Diego, CA).

## Results

### The Priming, but Not the Activating, Signal for IL-1β Production Is ROS-Dependent in Human Monocytes

**Figure 1:**
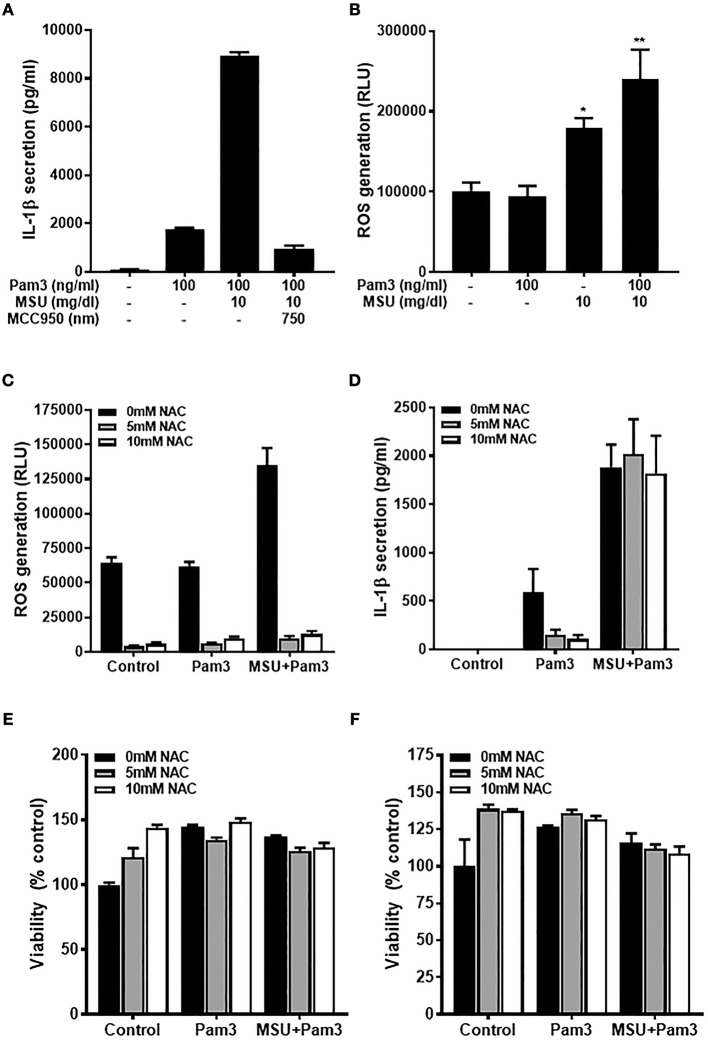
The priming but not activating signal is ROS-dependant in human monocytes. **(A)** IL-1β secretion from primary human monocytes. Cells were stimulated for 18 h with Pam3 and MSU crystals ± NLRP3 inhibitor MCC950. Bars represent means ± standard deviation and are representative of at least three independent experiments. **(B)** Primary human monocytes were stimulated for 2 h with Pam3, MSU crystals, or Pam3 + MSU at the concentrations specified and ROS measured by ROS-Glo™ assay. **(C,D)** Primary human monocytes were stimulated for 6 h with Pam3 (100 ng/mL) ± MSU crystals (10 mg/dL) alongside the ROS inhibitor NAC (5–10 mM). ROS levels were measured by ROS-Glo™ assay **(C)** and IL-1β secretion was determined by ELISA **(D)**. Cell viability was determined at the end of the experiments shown in **(C,D)** by cell-titer Glo assay (**E,F**, respectively). Data represent means from 6 individual donors + SEM (*n* = 6). Significance was determined by Kruskall-Wallis analysis comparing treated vs. untreated controls (^*^P < 0.05, ^**^P < 0.01).

Primary human monocytes secrete IL-1β when stimulated with TLR ligands due to activation of the transcription factor NF-κB and subsequent increased transcription of the *IL1B* gene. In contrast to macrophages, the IL-1β produced is then secreted from monocytes via the constitutively active caspase-1 in these cells (40). Primary human monocytes secreted IL-1β when treated with the TLR2 ligand, Pam3, and these levels increased substantially when cells were treated with Pam3 + MSU (Figure 1A) via activation of the NLRP3 inflammasome, as demonstrated by inhibition of IL-1β secretion using NLRP3 inhibitor MCC950 (41). To investigate whether ROS are involved in IL-1β secretion, cells were treated with Pam3 –/+ crystallized uric acid and ROS levels measured by ROS-Glo^TM^ assay. Crystallized uric acid (MSU), but not Pam3, resulted in increased ROS levels in these cells (Figure 1B).

The fact that MSU increased ROS in human monocytes raised the possibility that this could be one mechanism by which crystallized uric acid could increase susceptibility to gout attacks by increasing the quantity of IL-1β secreted from monocytic cells. To test this hypothesis, human monocytes were treated with Pam3 –/+ MSU in the presence of NAC which reduced the concentration of ROS by more than 90% (Figure 1C). However, inhibiting ROS with NAC reduced the Pam3-induced IL-1β secretion, but had no effect on IL-1β secreted in response to Pam3 + MSU (Figure 1D). Treatment of the cells with NAC at concentrations of 5 mM or 10 mM had no effect on cell viability (Figures 1E,F), so the changes observed in IL-1β secretion were not due to cell death in these experiments.

### Activation of TLR2 Is Associated With Increased Gene Expression of *IL1B* and *SOD2* in Human Monocytes and Is Not Affected by Soluble Uric Acid

**Figure 2:**
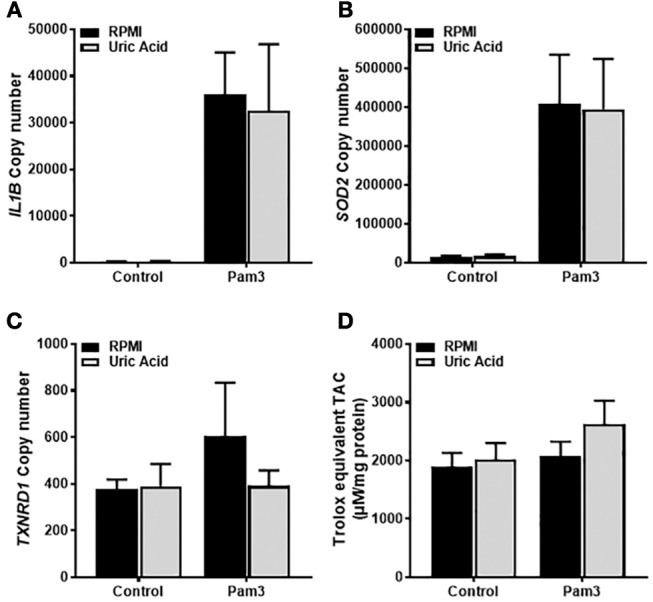
Gene expression of *IL1B* and *SOD2* in human monocytes is increased by TLR2 activation but unaffected by soluble uric acid. Primary human monocytes were stimulated for 6 h with Pam3 (100 ng/mL) following an initial exposure to culture media (RPMI) or soluble uric acid (30 mg/dL). Gene expression of *IL1B* **(A)**, *SOD2* **(B)**, and *TXNRD1* **(C)** were quantified by qPCR. **(D)** Human monocytes were stimulated as above for measurement of intracellular total antioxidant capacity (TAC) and expressed per mg of protein. Data represents means from 3 to 4 individual donors + SEM. Statistical analyses were performed by Mann-Whitney tests between control and uric acid treated samples in the presence of Pam3 (ns = not significant).

Given that the priming step of IL-1β production was ROS-dependent and that soluble uric acid has known antioxidant effects (24), we next tested whether exposure of monocytes to uric acid influenced IL-1β gene expression in response to Pam3. As expected, treatment with Pam3 resulted in a dramatic increase in the expression of *IL1B* (Figure 2A). Expression of the antioxidant genes *SOD2* and *TXNRD1* were also measured to test for any change in antioxidant response upon stimulation. Expression of *SOD2*, but not *TXNRD1*, was significantly increased in response to Pam3 (Figures 2B,C). Pre-incubation of the cells with 30 mg/dL (a concentration well in excess of clinical hyperuricemia) had no effect on the expression levels of *IL1B, SOD2*, or *TXNRD1* (Figures 2A–C). In addition, there were no changes in the total intracellular antioxidant capacity in response to Pam3 –/+ uric acid (Figure 2D).

### Alterations in Serum Antioxidant Capacity of Gout Patients

Uric acid is the major antioxidant in the blood; therefore hyperuricemia could result in alterations in the extracellular redox environment that could impact on the responsiveness of monocytes to priming and activation of the NLRP3 inflammasome. Sera from gout patients were analyzed to determine uric acid concentration together with two of the major extracellular antioxidant enzymes, ecSOD and Gpx3, and the TAC. Blood samples were also collected from patients with CKD and from patients with RA for comparison.

**Figure 3:**
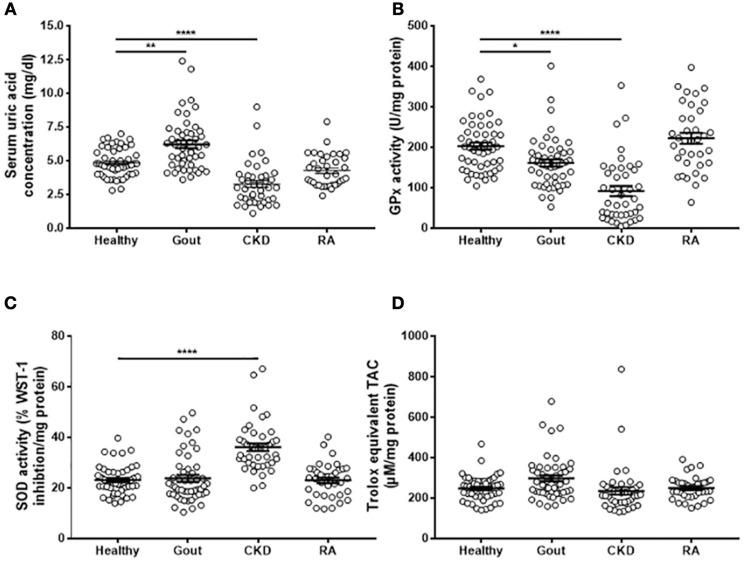
Serum antioxidant capacity in gout, CKD and RA patients. Serum was isolated from whole blood of healthy controls (*n* = 52), gout (*n* = 50), CKD (*n* = 42), or RA (*n* = 36) patients. **(A)** Uric acid concentration was determined using amplex-red based uric acid assays. **(B)** GPx and **(C)** SOD activities were measured using kinetic enzyme assays. **(D)** TAC was measured by TAC assay kit and quantified by reference to a Trolox standard curve. Serum enzyme activities and TAC ware expressed as activity per mg protein. Individual points represent means for 2–3 replicate measurements for each donor. Error bars represent means ± SEM of the entire population. Significance was determined by Kruskall–Wallis analysis comparing disease vs. healthy controls (^*^P < 0.05, ^**^P < 0.01, ^****^P < 0.0001).

Gout patients had significantly greater serum uric acid concentrations than controls, while CKD patients had lower serum uric acid (Figure 3A). Sera from both gout and CKD patients had significantly reduced GPx activity compared to healthy controls (Figure 3B), and CKD patients had increased SOD activity (Figure 3C). This could be a compensatory measure as there were no differences in overall TAC detected between the four patient groups (Figure 3D). No significant differences in antioxidant capacity were detected in sera from RA patients, so these samples were not subject to further analysis.

### Monocytes From Gout Patients Secrete Higher Levels of IL-1β in Response to Pam3 Than Healthy Controls

**Figure 4:**
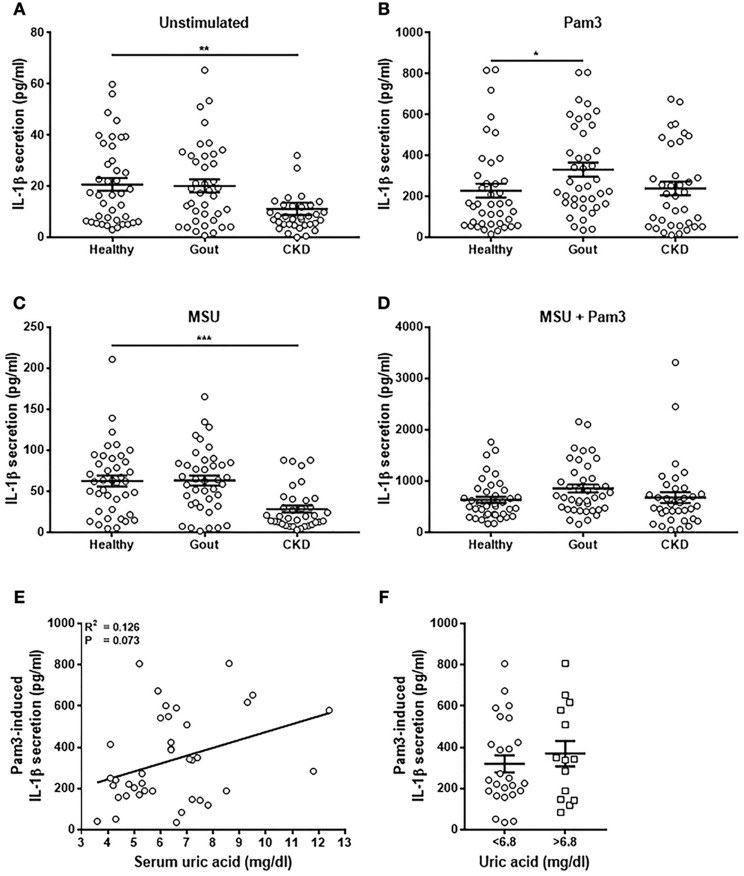
Monocytes from gout patients secrete higher levels of IL-1β in response to Pam3, but there is no correlation between IL-1β production and serum uric acid concentration. Monocytes were isolated from whole blood from healthy (*n* = 40), gout (*n* = 40), or CKD (*n* = 37) donors and stimulated for 18 h with cell culture media **(A)**, Pam3 (100 ng/mL) **(B)**, MSU (10 mg/dL) **(C)**, or a combination of MSU and Pam3 **(D)**. IL-1β secretion was determined by ELISA. **(E)** Correlation of IL-1β secretion from monocytes and serum uric acid concentration in gout patients. Each point represents means from 3 to 6 replicate measurements. **(F)** IL-1β secretion from monocytes from gout patients was analyzed by grouping samples according to serum uric acid concentration. Hyperuricemia is represented by a serum uric acid of ≥6.8 mg/dL and normouricemia by serum uric acid of ≤ 6.8 mg/dL. Error bars represent mean ± SEM. Individual points represent means from 3 to 6 replicate measurements for each donor. Error bars represent means ± SEM of the entire population. Significance was determined by Kruskall-Wallis analysis comparing disease vs. healthy controls (^*^P < 0.05, ^**^P < 0.01, ^***^P < 0.001).

Monocytes were isolated from patient blood and stimulated *in vitro* with Pam3, MSU, or a combination of Pam3 and MSU and IL-1β production measured by ELISA. Secretion of IL-1β was significantly lower from monocytes from CKD patients when unstimulated (Figure 4A) and when treated with MSU (Figure 4C). Monocytes isolated from gout patients secreted significantly greater amounts of IL-1β in response to Pam3 compared with monocytes from healthy controls (Figure 4B). However, there was no correlation between the quantity of IL-1β secreted and serum uric acid concentration (Figure 4E) even when samples were divided into normouricaemic vs. hyperuricemic (Figure 4F). These terms refer to clinically defined normal serum uric acid concentration (normouricemic; ≥6.8 mg/dL) or high serum uric acid concentration (hyperuricemic; ≤ 6.8 mg/dL). There were no differences in the quantities of IL-1β secreted from monocytes isolated from any of the groups in response to Pam3 + MSU (Figure 4D).

### Expression of Genes Coding for Components of the NLRP3 Inflammasome Are Unchanged in Monocytes From Gout Patients

**Figure 5:**
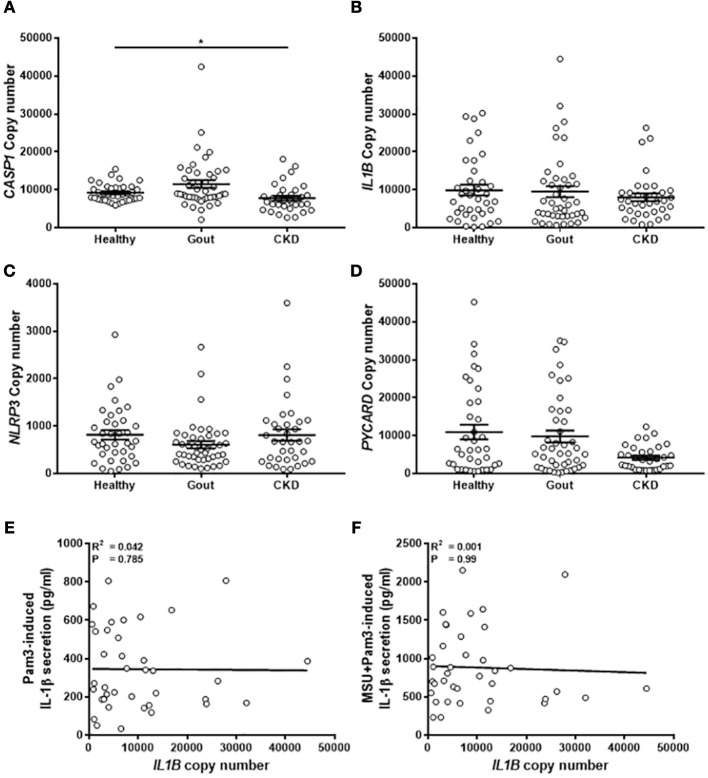
Expression of genes coding for components of the NLRP3 inflammasome are unchanged in monocytes from gout patients. cDNA was prepared from human monocytes from healthy (*n* = 37), gout (*n* = 43), or CKD (*n* = 35–37) donors. Expression of *CASP1* **(A)**, *IL1B* **(B)**, *NLRP3* **(C)**, and *PYCARD* **(D)** was measured by absolute qPCR. Copy numbers were calculated using standard curves for each gene of interest and normalized to expression of the reference genes *B2M* and *RPL32*. Individual points represent means from 2 to 3 replicate measurements for each donor and error bars represent means ± SEM. Significance was determined by Kruskall-Wallis (^*^P < 0.05). Correlation of *IL1B* gene expression in monocytes from gout patients with IL-1β secretion in response to **(E)** Pam3 or **(F)** Pam3 + MSU crystals. Each point represent means from 3 to 6 replicate measurements for each donor. Error bars represent mean of entire population ± SEM. Correlation significance was determined by Spearman correlation test.

The expression of components of the NLRP3 inflammasome (pro-IL-1β, NLRP3, pro-caspase-1, ASC-1; gene name *PYCARD*) was measured by absolute qPCR to determine if IL-1β production in the monocytes was determined by expression of these genes. There were no differences in gene expression between the different populations of monocytes tested (Figure 5), apart from lower levels of expression of caspase-1 in the monocytes isolated from CKD patients (Figure 5A). Expression of *PYCARD* was also lower in these cells but did not reach statistical significance (Figure 5D). Intriguingly, there was no correlation between expression of *IL1B* in monocytes from gout patients and concentration of IL-1β secreted from these cells in response to any stimulus (Figures 5E,F).

### Monocytes Isolated From Gout Patients Express Higher Levels of *TXNRD1* but Total Intracellular Antioxidant Capacity Is Unchanged

**Figure 6:**
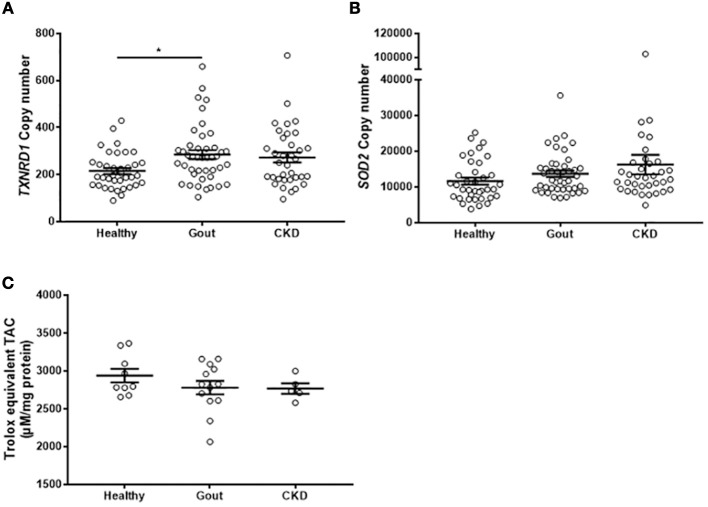
Monocytes from gout patients express higher levels of *TXNRD1* but overall total intracellular antioxidant capacity is unchanged. cDNA was prepared from monocytes isolated from healthy (*n* = 37), gout (*n* = 43), or CKD (*n* = 37) donors. Expression of *TXNRD1* **(A)** and *SOD2* **(B)** was calculated by absolute qPCR. Copy numbers were calculated using standard curves for each gene of interest and normalized to expression of reference genes *B2M* and *RPL32*. **(C)** Monocytes from healthy (*n* = 9), gout (*n* = 13), or CKD (*n* = 5) donors were lysed in NP40 cell lysis buffer and intracellular TAC and protein content were measured. Individual points represent means from 2 to 3 replicate measurements for each donor. Error bars represent means ± SEM of the entire population. Significance was determined by Kruskall-Wallis comparing disease vs. healthy controls (^*^P < 0.05).

The analyses of gene expression in monocytes isolated from gout patients did not show any difference from healthy control monocytes. However, exposure to high concentrations of uric acid as would occur in hyperuricemic patients, could lead to alterations in the intracellular redox environment. To test whether there are any changes in the expression of intracellular antioxidant defenses, expression of intracellular *SOD2* and *TXNRD1* were measured in the monocytes isolated from patients, as well as the total intracellular antioxidant capacity. There was increased expression of *TXNRD1* in the monocytes from gout patients (Figure 6A), but no change in expression of *SOD2* (Figure 6B) or of TAC (Figure 6C).

## Discussion

Many studies have proposed a mechanistic link between IL-1β production and increases in ROS or oxidative stress (8, 14, 17, 42). The results of this study do not support such a premise, at least not as a direct or indeed necessary effect in peripheral blood monocytes. However, we cannot rule out the possibility that increases in ROS and/or oxidative stress play a role in IL-1β production from tissue-resident macrophages during a gout attack. There are well-known differences in the signaling pathways involved in the production and processing of IL-1β between monocytes and macrophages, not least in the absolute requirement for a second signal via ATP stimulation in addition to TLR ligation (40). Canonical NLRP3 inflammasome activation requires both priming and activating signals. Pam3 can function as a priming signal via ligation of TLR2, activating NF-κB, and inducing transcription of pro-IL-1β and NLRP3 (43). A second signal such as MSU is required for oligomerisation of NLRP3, ASC and pro-caspase-1 which then leads to the processing and secretion of IL-1β (44). The results shown here are consistent with this canonical signaling mechanism, with a dramatic increase in IL-1β secretion in the presence of MSU.

Although MSU increased ROS in primary human monocytes, this was not the mechanism by which MSU increased IL-1β secretion from these cells as inhibiting ROS had no effect on IL-1β secretion. Our results suggest that if oxidative stress has any effect on IL-1β production, it is only on the priming part of the pathway and not on activation of NLRP3, consistent with some previous studies (45, 46) but not with others (8, 47). Firstly, inhibition of ROS using NAC inhibited Pam3-induced IL-1β secretion but not Pam3+MSU-induced IL-1β. Secondly, monocytes isolated from gout patients were more responsive to Pam3, but not to Pam3+MSU where the levels of IL-1β secreted were similar to those from healthy control monocytes.

Our findings are in agreement with a recent study showing increased IL-1β secretion in PBMCs from gout patients compared to healthy donors in response to Pam3 (48). However, in contrast to our findings, this earlier work also found increased IL-1β secretion from PBMCs in response to Pam3+MSU (48). These differences could be due to the use of human monocytes, rather than PBMCs where monocytes account for about 8–10% of the total. There is some constitutively active caspase-1 in human monocytes which is why IL-1β is secreted in response to Pam3 alone (40) with further cleavage of pro-caspase-1 as a consequence of NLRP3 oligomerization.

There were no differences in gene expression of *NLRP3, PYCARD*, or *CASP1* in monocytes from people with gout which was consistent with our observations that pre-incubation of monocytes with uric acid did not influence gene expression of *IL1B* or the antioxidant enzymes *SOD2* or *TXNRD1*. This suggests that susceptibility to gout attacks is not due to an inherent hypersensitivity in the leukocytes of these patients nor is there a compensatory increase in expression of SOD2 that might reflect increased oxidative stress. There is nothing in these data to suggest that exposure of monocytes to uric acid which, by definition, occurs at least some of the time in people with gout, has any effect on subsequent monocyte responses to Pam3+MSU. This is particularly interesting given that uric acid can increase ROS levels in many cell types including hepatocytes (49), adipocytes (50), vascular smooth muscle cells (51), and THP-1 monocytes (52). In addition, we also observed increases in ROS in response to uric acid in primary human monocytes (manuscript in revision). However, the data presented here show that any increases in ROS that occur in response to uric acid do not appear to be associated with changes in IL-1β expression, processing, or secretion. A limitation of this study is that we did not have sufficient material from the patient samples to allow analyses of the protein levels of the components of the NLRP3 inflammasome. Although no differences in expression of these genes as measured by qPCR were observed, there may be differences in the levels of these proteins due to differences in their translation. Further studies are required to address this possibility.

It is interesting to note that although uric acid increased the production of ROS in monocytes, it did not appear to impact on the overall level of oxidative stress in the cells. In blood from gout patients where higher levels of uric acid were measured, there were lower levels of other antioxidants and this may have resulted in maintaining the overall antioxidant capacity at appropriate levels. Blood from patients with CKD contained significantly lower concentrations of uric acid and, similar to previous studies, of Gpx3 (53, 54), but higher activity levels of the antioxidant enzyme ecSOD. It is not clear whether these different antioxidants are able to compensate for each other *in vivo*, particularly as they detoxify different types of ROS. It is interesting that there were no changes in the overall antioxidant capacity in any of groups tested despite differences in serum uric acid. This ability to buffer against changes in oxidative stress points to the presence of many mechanisms that operate in concert to control redox status and explains why changes in serum uric acid alone is not sufficient to cause release of IL-1β and contribute to development of gout.

There was no correlation between expression and secretion of IL-1β either in response to Pam3 or Pam3+MSU. This is not perhaps so surprising as it has been known for some time that synthesis of IL-1-β is regulated by both the level of transcription and translational efficiency (55). Previous studies have also shown that TLR2 ligands can result in increased synthesis and intracellular processing of IL-1β but without significant IL-1β release and notably, this work was done in primary human monocytes (56). It is difficult to explain why this would be the case here, particularly in the presence of MSU which causes activation of the NLRP3 inflammasome. Further work is necessary to determine the mechanism by which increased expression of IL-1β fails to translate into increased secretion of the active cytokine, with a particular focus on whether activation of the NLRP3 inflammasome can be un-coupled from release of IL-1β.

A striking feature of these results is the lack of correlation between serum uric acid concentration and production of IL-1β in monocytes from gout patients, given that hyperuricaemia is the single greatest risk factor for gout. This relationship, or lack thereof, was confirmed by the low serum uric acid levels in CKD patients which had no effect on the ability of these monocytes to respond to Pam or Pam3+MSU. Previous studies have shown that leukocytes from patients with CKD are hyporesponsive in terms of cytokine secretion (57) and we did observe less IL-1β secretion from the cells isolated from CKD patients here, but only in basal (unstimulated) conditions and in response to MSU alone.

The only difference in production of IL-1β was in the monocytes from gout patients in response to Pam3. However, this phenomenon also appears to be independent of exposure of these cells to uric acid. Therefore, there must be additional, as yet unknown, factors that regulate the production of IL-1β that are independent of intercellular or extracellular antioxidant status.

## Author Contributions

BA acquired, analyzed, and interpreted data as well as developed study concept and wrote manuscript. CB acquired data. KB, PG, and KD contributed to study conception and design. LM developed the study concept, obtained funding and ethics, interpreted data, and wrote manuscript. All authors read and approved the final manuscript.

### Conflict of Interest Statement

The authors declare that the research was conducted in the absence of any commercial or financial relationships that could be construed as a potential conflict of interest.
